# Differentiation of Francisella tularensis Subspecies and Subtypes

**DOI:** 10.1128/JCM.01495-19

**Published:** 2020-03-25

**Authors:** Marilynn A. Larson, Khalid Sayood, Amanda M. Bartling, Jennifer R. Meyer, Clarise Starr, James Baldwin, Michael P. Dempsey

**Affiliations:** a Department of Pathology and Microbiology, University of Nebraska Medical Center, Omaha, Nebraska, USA; b National Strategic Research Institute, Omaha, Nebraska, USA; c Department of Electrical Engineering, University of Nebraska-Lincoln, Lincoln, Nebraska, USA; d Air Force Research Laboratory, Applied Technology and Genomics Division, Wright-Patterson Air Force Base, Ohio, USA; Johns Hopkins University School of Medicine

**Keywords:** *Francisella tularensis*, tularemia, subspecies and subtype differentiation, genotyping, subspeciation, singleplex and multiplex quantitative real-time PCR, diagnostics

## Abstract

The highly infectious and zoonotic pathogen Francisella tularensis is the etiologic agent of tularemia, a potentially fatal disease if untreated. Despite the high average nucleotide identity, which is >99.2% for the virulent subspecies and >98% for all four subspecies, including the opportunistic microbe Francisella tularensis subsp. novicida, there are considerable differences in genetic organization. These chromosomal disparities contribute to the substantial differences in virulence observed between the various F. tularensis subspecies and subtypes. The methods currently available to genotype F. tularensis cannot conclusively identify the associated subpopulation without using time-consuming testing or complex scoring matrices. To address this need, we developed both single and multiplex quantitative real-time PCR (qPCR) assays that can accurately detect and identify the hypervirulent F. tularensis subsp. tularensis subtype A.I, the virulent F. tularensis subsp. *tularensis* subtype A.II, F. tularensis subsp. holarctica (also referred to as type B), and F. tularensis subsp. mediasiatica, as well as opportunistic F. tularensis subsp. *novicida* from each other and near neighbors, such as Francisella philomiragia, Francisella persica, and *Francisella*-like endosymbionts found in ticks. These fluorescence-based singleplex and non-matrix scoring multiplex qPCR assays utilize a hydrolysis probe, providing sensitive and specific F. tularensis subspecies and subtype identification in a rapid manner. Furthermore, sequencing of the amplified F. tularensis targets provides clade confirmation and informative strain-specific details. Application of these qPCR- and sequencing-based detection assays will provide an improved capability for molecular typing and clinical diagnostics, as well as facilitate the accurate identification and differentiation of F. tularensis subpopulations during epidemiological investigations of tularemia source outbreaks.

## Abstract (precis)

The highly infectious and zoonotic pathogen Francisella tularensis is the etiologic agent of tularemia, a potentially fatal disease if untreated. Despite the high average nucleotide identity, which is >99.2% for the virulent subspecies and >98% for all four subspecies, including the opportunistic microbe Francisella tularensis subsp. novicida, there are considerable differences in genetic organization. These chromosomal disparities contribute to the substantial differences in virulence observed between the various F. tularensis subspecies and subtypes.

## INTRODUCTION

Tularemia is a zoonotic disease caused by Francisella tularensis, a facultative intracellular pathogen that may be easily disseminated with a lethal dose of less than 10 organisms (1). As such, F. tularensis is classified as a tier 1 select agent and potential bioweapon (2). Select agents have the potential to pose a severe threat to public health and safety, and the microbes designated a tier 1 select agent present the greatest risk for deliberate misuse, causing mass casualties and devastating effects to the economy and infrastructure (https://www.selectagents.gov/bbp-definitions.html). All select agents are regulated by the Centers for Disease Control and Prevention (CDC) Federal Select Agent Program (FSAP).

F. tularensis is comprised of four subspecies, specifically F. tularensis subsp. tularensis (type A), F. tularensis subsp. holarctica (type B), F. tularensis subsp. mediasiatica, and F. tularensis subsp. novicida, of which the latter classification in this species is controversial (3, 4). The type A strains are further separated into subtypes A.I and A.II. Importantly, these subspecies and subtypes differ considerably in virulence, with the subtype A.I clade being the most virulent (5).

Three of the four F. tularensis subspecies, specifically F. tularensis subsp. *tularensis*, F. tularensis subsp. *holarctica*, and F. tularensis subsp. *mediasiatica* are considered select agents, whereas F. tularensis subsp. *novicida* is not a select agent, along with the attenuated strains B-38 (ATCC 6223), LVS, and SCHU S4 Δ*clpB* (https://www.selectagents.gov/exclusions-hhs.html). The chromosome of the select agent F. tularensis subspecies (F. tularensis subsp. *tularensis*, F. tularensis subsp. *holarctica*, and F. tularensis subsp. *mediasiatica*) have a duplicated *Francisella* pathogenicity island (FPI) and numerous insertion sequence (IS) elements. In contrast, the genome of opportunistic F. tularensis subsp. *novicida* contains a single FPI and just a few IS elements but still causes indeterminate identification results with some diagnostic platforms due to a high average nucleotide identity with the select agent strains.

F. tularensis subtype A.I, type B, and F. tularensis subsp. *novicida* strains are distributed throughout the United States, with type B and F. tularensis subsp. *novicida* strains being endemic throughout the Northern Hemisphere and Eurasia (6, 7). F. tularensis subtype A.II strains appear to be geographically associated with the western regions of the United States, and F. tularensis subsp. *mediasiatica* strains have been isolated from Central Asian Republics in the former Union of Soviet Socialist Republics (USSR) (8, 9). The hypervirulent F. tularensis A.I strains are among the most pathogenic bacteria known, but all the clades can infect numerous species (>250) and persist in the environment (10). Known reservoirs of F. tularensis include infected animals, arthropod vectors (e.g., ticks, mosquitoes, and deer flies), soil, food, and water. Common modes of tularemia transmission can occur through inhalation and ingestion, as well as cutaneous and conjunctival routes of exposure to this pathogen. Inhalation of contaminated aerosols causes the most severe form of this disease, specifically pneumonic tularemia. However, all forms of tularemia if untreated can lead to hematogenous spread and eventual acute renal failure (11, 12). Fatality rates as high as 35% from a subtype A.I infection have been reported (6).

From a public health and Department of Defense force health protection perspective, fast and accurate identification of exposure to a virulent F. tularensis strain, particularly a subtype A.I strain, is imperative. For all exposures, determination of the F. tularensis subspecies and subtype is critical to ensure that appropriate medical management occurs, particularly for the virulent clades (13); however, this capability is currently lacking. Public health laboratories are in need of a fast and accurate assay that can definitively distinguish the virulent select agent F. tularensis subspecies (F. tularensis subsp. *tularensis*, F. tularensis subsp. *holarctica*, and F. tularensis subsp. *mediasiatica*) from the opportunistic F. tularensis subsp. *novicida*, which is not a select agent. Furthermore, a number of other environmental microbes share genomic content with F. tularensis, complicating the accurate identification of select agent F. tularensis strains from near neighbors, such as the nonpathogenic tick endosymbiont *F. persica* (formally classified as Wolbachia persica) (14).

Notable progress has been made in the field of infectious disease diagnostics. A PCR assay that is based on the different chromosomal locations of insertion sequence elements accurately identifies F. tularensis to the subspecies and subtype level; however, this method requires visualization of the resulting amplicons by gel fractionation and subsequent staining (15). Although there are valuable quantitative real-time PCR (qPCR) assays available to identify F. tularensis, these rapid methodologies do not differentiate the four subspecies and two subtypes. For example, a qPCR assay capable of identifying F. tularensis to the subspecies level has been described, but this platform requires a complex scoring matrix and is only able to differentiate three of the four F. tularensis subspecies with moderate sensitivity (16). Others have developed singleplex qPCR assays that targeted 11 different single nucleotide polymorphisms (SNPs) that were potentially specific to closely related groups within the genus *Francisella*, including some of the F. tularensis subpopulations (17). To obtain dependable results, however, parameter modulation in several of these assays was required, as well as a stepwise hierarchical scheme for interpretation. Sporadic amplification failure or nonspecific amplification was also stated to unpredictably occur, which was attributed to additional SNPs within the primer/probe sites but was never confirmed.

In this report, we describe rapid, accurate, and sensitive stand-alone singleplex and two non-matrix scoring multiplex qPCR detection assays that can differentiate all four F. tularensis subspecies and the two subtypes, as well as a sequencing-based method, confirming clade identity and providing informative strain-specific details. These flexible next-generation platforms are suitable for use by public health laboratories, including the CDC and other agencies and institutes, replacing existing tests that lack these capabilities.

## MATERIALS AND METHODS

### Bacterial strains and ticks.

Wild-type and reference strains of F. tularensis were obtained from multiple sources, including Biodefense and Emerging Infections Research (BEI) Resources Repository (Manassas, VA) and public health laboratories in the United States. Select agent strains were transported to the University of Nebraska Medical Center/Nebraska Public Health Laboratory in Omaha following the requirements of the FSAP, as outlined in the Animal and Plant Health Inspection Service/CDC Form 2, Guidance Document for Request to Transfer Select Agents and Toxins (18). Manipulation of viable culture material was performed by authorized individuals within the biosafety level 3 facility at the University of Nebraska Medical Center that is certified for select agent work by the FSAP. Appropriate laboratory biosafety criteria was utilized, as described by the National Institutes of Health and CDC (19). The non-select agent bacterial strains were obtained from the American Type Culture Collection (ATCC; Manassas, VA), BEI Resources, or the Nebraska Public Health Laboratory.

Species-level identification of all F. tularensis isolates was confirmed following the testing protocols used by the Laboratory Response Network reference laboratories. In addition, pulsed-field gel electrophoresis (PFGE) with the restriction endonucleases PmeI and BamHI verified the subpopulation classification the F. tularensis reference strains and wild-type isolates, which was previously described (15). The F. tularensis inclusivity panel consisted of 10 F. tularensis subsp. *tularensis* subtype A.I strains, 10 F. tularensis subsp. *tularensis* subtype A.II strains, 10 F. tularensis subsp. *holarctica* (type B) strains, 3 F. tularensis subsp. *mediasiatica* strains, and 2 F. tularensis subsp. *novicida* strains (Table 1). The F. tularensis reference strains in this test panel included SCHU S4 (F. tularensis subsp. *tularensis* subtype A.I), WY96-3418 (F. tularensis subsp. *tularensis* subtype A.II), LVS (F. tularensis subsp. *holarctica*, type B), FSC147 (F. tularensis subsp. *mediasiatica*), and U112 (F. tularensis subsp. *novicida*). To assess specificity, the exclusivity panel was comprised of bacterial species other than F. tularensis, including Francisella philomiragia and Francisella persica, as well as ticks that are known to harbor uncharacterized F. tularensis-like endosymbionts (Table 2 and 3). Ticks were collected from various animals, as well as by flagging or dragging, and were identified to the species level using published keys (Table 3) (20, 21). To verify the presence of F. tularensis-like endosymbionts in the ticks, PCR amplification was performed using a commercial assay that is no longer available, along with previously described primers that target conserved genes in these organisms and F. tularensis (22–25).

**TABLE 1:** Francisella tularensis subspecies, types or subtypes, and strains used in this study for the inclusivity test panel^*a*^

Type or subtype by subspecies	Strain designation(s)	Geographic origin	Infected host or source	Yr isolated
F. tularensis subsp. tularensis				
A.I	SCHU S4	Ohio	Human	1941
A.I	NE-NPHL-061598	Nebraska	Human	1998
A.I	OK-OSU-98041035	Oklahoma	Cat	1998
A.I	NC-RADDL-48620-97	North Carolina	Rabbit	1997
A.I	AK-APHL_1100558	Arkansas	Hare	2004
A.I	MO-MPHL-D05	Missouri	Human	2005
A.I	NE-UNVDL-062807	Nebraska	Squirrel	2007
A.I	WY-WPHL-06F12348	Wyoming	Human	2006
A.I	NE-Child-090712	Nebraska	Human	2012
A.I	NE-UNLVDL-070213	Nebraska	Cat	2013
A.II	WY96-3418, NR-644^*b*^	Wyoming	Human	1996
A.II	WY-WSVL-00W4114	Wyoming	Prairie dog	2000
A.II	UT-UDHL-80402860	Wyoming	Human	2004
A.II	WY-WPHL-BT324	Wyoming	Human	1996
A.II	ATCC 6223	Unknown	Unknown	Unknown
A.II	WY-WPHL-03W10146	Wyoming	Human	2003
A.II	WY-WPHL-05W9954	Wyoming	Human	2005
A.II	WY-WPHL-06W9410	Wyoming	Human	2006
A.II	UT-UDHL-70102163	Utah	Human	2001
A.II	WY-WPHL-07F13554	Wyoming	Human	2007
F. tularensis subsp. holarctica				
B	LVS	Russia	Vole	Unknown
B	WY-WSVL-96194280	Wyoming	Rabbit	1996
B	WY-WSVL-9868529	Wyoming	Guinea pig	1998
B	WY-WSVL-OvineNC	Wyoming	Sheep	Unknown
B	FR-LR	France	Human	1993
B	NE-NPHL-061705	Nebraska	Human	2005
B	MO-MPHL-G05	Missouri	Human	2005
B	UT-UDH-70001092	Utah	Human	2000
B	NE-NPHL-072606	Nebraska	Human	2006
B	NE-Methodist-061113	Nebraska	Human	2013
F. tularensis subsp. mediasiatica				
N/A	FSC147^*b*^	Kazakhstan	Gerbil	1965
N/A	FSC148	Central Asia	Tick	1982
N/A	FSC149	Central Asia	Unknown	Unknown
F. tularensis subsp. novicida				
N/A	U112	Utah	Water	1951
N/A	NE-NPHL-101315	Nebraska	Human	2015

a LVS, live vaccine strain; N/A, not applicable.

b Reference strains SCHU S4 (F. tularensis subsp. *tularensis* subtype A.I), WY96-3418 (F. tularensis subsp. *tularensis* subtype A.II), LVS (F. tularensis subsp. *holarctica*, also known as type B), FSC147 (F. tularensis subsp. *mediasiatica*), and U112 (F. tularensis subsp. *novicida*) were used for the initial screening process.

**TABLE 2:** Species and strains of bacteria used in this study for the exclusivity test panel

Bacterial species	Strain	Bacterial class
Francisella philomiragia	ATCC 25015	*Gammaproteobacteria*
Francisella persica	ATCC VR-331	*Gammaproteobacteria*
Escherichia coli	ATCC 35218	*Alphaproteobacteria*
Pseudotuberculosis aeruginosa	ATCC 27853	*Gammaproteobacteria*
Yersinia pseudotuberculosis	6902	*Gammaproteobacteria*
Haemophilus influenza	ATCC 12011	*Gammaproteobacteria*
Acinetobacter baumannii	ATCC 19606	*Gammaproteobacteria*
Legionella pneumophilia	ATCC 33152	*Gammaproteobacteria*
Burkholderia cepacia	ATCC 25608	*Gammaproteobacteria*
Bacillus anthracis	Sterne	*Betaproteobacteria*

**TABLE 3:** Environmental ticks included in the exclusivity test panel, along with associated details

Tick species	Tick isolate designation	Geographic origin	Infected host or source	Tick sex	Presence of FLE(s)^*a*^
Dermacentor andersoni	T19	South Dakota	Cow	Female	No
Dermacentor andersoni	T58	South Dakota	Cow	Male	Yes
Dermacentor andersoni	T78	South Dakota	Field	Male	ND
Dermacentor variabilis	T143	Iowa	Field	Female	Yes
Dermacentor variabilis	T144	Iowa	Field	Male	Yes
Dermacentor variabilis	T161	Virginia	Field	Female	Yes
Dermacentor variabilis	T163	Virginia	Field	Female	Yes
Dermacentor variabilis	T165	Virginia	Field	Male	Yes
Amblyomma americanum	T169	Virginia	Field	Female	No
Amblyomma americanum	T172	Virginia	Field	Male	No
Ixodes scapularis	T173	Virginia	Field	Male	No
Amblyomma americanum	T194	Iowa	Dog	Male	No
Amblyomma americanum	T206	Iowa	Dog	Female	No
Amblyomma americanum	T211	Iowa	Field	Female	No
Dermacentor andersoni	T224	Montana	Field	Female	Yes
Dermacentor andersoni	T225	Montana	Field	Male	Yes
Dermacentor andersoni	T243	Montana	Field	Male	ND
Ixodes scapularis	T277	Montana	Dog	Male	No
Ixodes scapularis	T278	Montana	Dog	Female	No
Ixodes scapularis	T279	Montana	Dog	Female	No
Dermacentor variabilis	T382	Nebraska	Field	Female	ND
Dermacentor variabilis	T404	Iowa	Dog	Female	ND
Dermacentor variabilis	T405	Nebraska	Dog	Female	ND
Dermacentor variabilis	T413	Iowa	Dog	Male	Yes
Dermacentor variabilis	T417	Nebraska	Field	Male	ND

a FLE, *Francisella*-like endosymbiont; ND, not determined.

F. tularensis strains were subcultured on commercially available chocolate agar plates (catalog number R01302; Remel, Lenexa, KS) and were incubated at 37°C with 5% CO_2_ for 3 days before processing. Bacterial species other than F. tularensis were cultured in brain heart infusion broth, with the exception of Legionella pneumophilia ATCC 33152 and F. persica ATCC VR-331. *L. pneumophilia* was grown in buffered charcoal-yeast extract medium, and *F. persica* ATCC VR-331 was cultured in complex medium as previously described (14, 26).

### DNA isolation and quantification.

Genomic DNA from the Gram-negative and Gram-positive bacteria assessed in this study was extracted using the Gentra Puregene yeast/bacteria kit (Qiagen, Valencia, CA) and the MasterPure Gram-positive DNA purification kit (Lucigen, Middleton, WI), respectively, as recommended by the associated manufacturer. F. tularensis genomic DNA was also isolated using cetyltrimethylammonium bromide (CTAB) according to standard procedures (27), and assay performance was equally robust regardless of the template DNA preparation method utilized. The environmental tick samples were homogenized in cold phosphate-buffered saline (PBS) using a Mini-Beadbeater-8 (BioSpec Products Inc., Bartlesville, OK), according to the manufacturer’s instructions. DNA was then isolated from the tick extracts using the QIAamp DNA blood mini kit (Qiagen) as recommended by the manufacturer, except that the 56°C incubation was performed overnight instead of for only 10 minutes. The tick DNA was concentrated, and 100 pg was utilized in the PCR-based assays. Isolated DNA was quantified using a NanoDrop UV/visible spectrophotometer (Thermo Fisher Scientific, Waltham, MA) and Qubit fluorometer (Promega, Madison, WI). DNA purity was determined by assessing the *A*_260_/*A*_280_ ratio, and genomic DNA quality was evaluated by visualization on an agarose gel, after fractionation and nucleic acid staining.

### *In silico* analyses.

Computational biology was used to develop a distance matrix algorithm that identified unique 21-mer genomic signatures for the various F. tularensis subpopulations. These signatures were then used to generate a list of potential targets for the subtype and subspecies identification assays. Candidate targets were further evaluated *in silico* for prospective use in the F. tularensis differentiation qPCR assays by utilizing the Basic Local Alignment Search Tool (BLAST) (https://blast.ncbi.nlm.nih.gov/Blast.cgi) to verify nucleotide uniqueness or shared homology for the prospective F. tularensis target in the appropriate subpopulations. These *in silico* analyses utilized every available nucleotide sequence in all public databases, including assembled genomes, draft genomes, and short sequence reads. This process was repeated until a targeted region of 60 to 250 bp in length fulfilled the required specificity. The Clustal Omega multiple sequence alignment program (https://www.ebi.ac.uk/Tools/msa/clustalo/) was then utilized to facilitate the designing of a primer pair with a hydrolysis probe for the target region in the appropriate and all available F. tularensis strains for subsequent testing in PCR-based assays.

### PCR screening of candidate targets.

F. tularensis-specific forward and reverse primers were used in conventional PCR for the initial evaluations. These initial PCR assessments utilized the five F. tularensis reference strains (SCHU S4, WY96-3418, LVS, FSC147, and U112), as well as *F. philomiragia* ATCC 25015, *F. persica* VR-331, and Escherichia coli ATCC 35218. For PCR screening of the candidate targets, Platinum DNA polymerase (Invitrogen, Carlsbad, CA) at a concentration of 0.5 units was used along with 1 ng of the appropriate template, 2 mM MgCl_2_, 1× PCR buffer, 0.25 mM each dNTP, and 0.5 μM each primer in a 25-μl reaction. No-template controls (NTCs), which do not containing any target DNA, were included to monitor for contamination and primer-dimer amplification products. Positive controls were included to ensure that the reaction components were functioning as expected. The PCR cycling parameters were 1 cycle for 2 min at 94°C and 35 cycles for 30 s at 94°C, 30 s at 54°C, and 30 s at 72°C, as recommended by the manufacturer. Resulting amplicons were fractionated in an agarose gel, stained with ethidium bromide, and visualized with UV light.

### Quantitative real-time PCR.

Hydrolysis probes with appropriate 5′ fluorophores and 3′ quenchers (Biosearch Technologies, Petaluma, CA) were designed for each of the top candidate targets for use with the 7500 Fast Dx real-time PCR system (Applied Biosystems, Waltham, MA) and 3M Integrated Cycler (Focus Diagnostics, Cypress, CA). The singleplex qPCR assays were initially performed with the F. tularensis reference strains using 0.2 μM of the appropriate hydrolysis probe labeled with a 5′ 6-carboxyfluorescein (FAM) fluorophore and a 3′ black hole quencher-1 (BHQ-1) quencher, 0.5 μM each appropriate primer pair, and 0.1 unit of Platinum DNA polymerase (Invitrogen) in a 20-μl reaction. The qPCR cycling parameters were 1 cycle for 2 min at 95°C and then 45 cycles for 1 s at 95°C and 20 s at 60°C. The primer pair and probe sets with the highest sensitivity and specificity were downselected for testing with genomic DNA from the inclusivity and the exclusivity test panel organisms.

The optimum forward and reverse primer concentrations were determined by using a 4 × 4 matrix that included concentrations of 0.25, 0.5, 0.75, and 1.0 μM for each primer. The optimum probe concentration was determined by evaluating duplicate amplification reaction mixtures containing final probe concentrations of 0.05, 0.1, 0.2, 0.3, 0.4, and 0.5 μM. The selected and optimized singleplex qPCR assays were then utilized in the development of multiplex qPCR platforms with the same qPCR cycling parameters. For the multiplex qPCR assays, the optimum concentration of each hydrolysis probe with an appropriate 5′ fluorophore and 3′ quencher for the qPCR instrument was again determined. These multiplex qPCR evaluations included final probe concentrations of 0.05, 0.1, 0.2, 0.3, 0.4, 0.5, 0.6, and 0.7 μM.

### Analytical sensitivity and specificity.

Standard curves were generated for the optimized singleplex and multiplex qPCR assays by using 10-fold serial dilutions of the appropriate F. tularensis reference genomic DNA. All dilutions were prepared in 50 mM Tris (pH 8.0) and at least in triplicate, with concentrations ranging from 1 ng to 0.1 fg. The R^2^ value obtained for each of the standard curves revealed the linearity of the assay, as well as confirmed repeatability and delineated the analytical sensitivity. Additional testing was performed as needed for more exactness on the limit of detection (LOD). To calculate the number of chromosomal copies for a particular concentration of F. tularensis genomic DNA being tested in the qPCR assay that amplifies a single-copy double-stranded DNA (dsDNA) target, the following calculation was used: copy number = (mass of dsDNA in grams/[bp length of dsDNA chromosome × 650 g/mol]) × (Avogadro’s number of ∼6.022 × 10^23^ molecules/mol).

After the LOD was determined for the singleplex and multiplex qPCR assays, DNA from each of the inclusivity test organisms was tested in duplicate at a 10-fold higher concentration than the LOD. To confirm analytical specificity, DNA from each of the exclusivity test organisms was tested at a 1,000-fold higher concentration than the LOD for each singleplex and multiplex qPCR assay. Different individuals in separate laboratories performed the singleplex and multiplex qPCR assays with the inclusivity and exclusivity samples in, at least, duplicates on various thermocyclers, confirming repeatability and reproducibility.

### Amplicon sequencing and data analysis.

To obtain F. tularensis strain-level information, amplicon sequencing was performed. The same primer sequences and cycling conditions were used as described above for the qPCR assays; however, the primers were synthesized with Personal Genome Machine (PGM)-compatible barcodes, as recommended in the Ion Amplicon Library Preparation (Fusion Method) User Guide (publication number 4468326, revision B). The Ion Xpress barcode adapters 1-16 kit (catalog number 4471250) was used, which provided 16 sets of primers and eliminated the need for library preparation. Amplification with these barcoded primers was performed on a Biometra TProfessional gradient thermocycler (Analytik Jena, Germany). The concentration of the resulting amplicons was determined by using the Ultrospec 2100 Pro UV/Vis spectrophotometer (Amersham Biosciences/GE Healthcare, Chicago, IL) and the 2100 bioanalyzer (Agilent, Santa Clara, CA), and amplicon sequencing was performed on a Ion Torrent PGM sequencer (Life Technologies, Carlsbad, CA), as recommended in the Ion PGM sequencing 400 kit manual. Amplicons were sequenced to a depth of greater than 20,000 reads per sample, and homology was evaluated using the National Center for Biotechnology Information BLAST and Reference Sequence (RefSeq) programs.

### Statistics.

Standard statistical analyses were performed using Microsoft Excel 2000 software for Windows. These analyses included determining the standard deviation for the different qPCR assays to quantify variability. An unpaired *t* test was used to compare qPCR assay sensitivity that was obtained by different individuals. Linear regression with best fit and R^2^ values were acquired for the standard curves obtained with each target in the qPCR assays to verify reproducibility and predictability.

## RESULTS

### Bacterial endogenous control for qPCR.

To determine if bacterial DNA was present and to serve as an endogenous internal control, the 16S rRNA gene was selected. The universal 16S rRNA gene primer and probe sequences were based on the sequences described by Yang et al. (28); however, the hydrolysis probe sequence was slightly modified to accommodate the *Francisella* genus and *Francisella*-like organisms. The resulting universal 16S rRNA primer pair and probe sequences are shown in Table 4, and the associated qPCR assay was referred to as U16S. The universal 16S rRNA gene primer pair was initially tested using conventional PCR to ensure the successful detection of the F. tularensis reference strains and the different bacterial species in the exclusivity panel. As expected, a 159-bp amplicon was produced for all the bacterial species evaluated.

**TABLE 4:** Primer pair and probe nucleotide sequences for associated qPCR assay with resulting amplicon size

Singleplex or multiplex qPCR assay	Amplicon size (bp)	Primers and associated probe identification	Primer (5′ to 3′)
U16S	159	U16s_forward primer	TGGAGCATGTGGTTTAATTCGA
		U16s_reverse primer	TGCGGGACTTAACCCAACA
		U16s_probe	CACGAGCTGACGACARCCRTGCA
4Pan1	116	4pan1_Forward primer	CAYCCTAGACTATTCTATACTTAC
		4pan1_Reverse primer	GTAAATCTATTTACTTGAAACATCTGC
		4pan1_Probe	CCGTACCAAGATCAAACAAATATACC
3Pan	83	3pan_Forward primer	TTTACACCCGTCTCCGTTAGT
		3pan_Reverse primer	CTCTTAAGGATGCAATTTGGGATT
		3pan_Probe	AAGAGGCAAAGCTGGAATTACACTCTCTC
A1d	114	A1d_forward primer	CACCCAGCAACAAAGTAGCAC
		A1d_reverse primer	CTATCTCATCATCAAAATCTATAAGAGC
		A1d_probe	CTCTTGCTGTTTTTTTAGCTGGATTATCC
A2c	101	A2c_forward primer	GGCTTTGCTAGCACAAATAAACC
		A2c_reverse primer	GATAAACAGCAATTCTTTAAGACGAC
		A2c_probe	CACTGTTAGTGACAATCCCTGCTATAG
B2	80	B2_forward primer	CCTATCCAATACTCCGAGTTAGT
		B2_reverse primer	AAATCAAAAGAAGAGTTAAAACAAGC
		B2_probe	CTCTGGCCAGTTATTTTTATCAAAGCCAG
M3	112	M3_forward primer	AGCACATGCTAGTTTAATGAGTT
		M3_reverse primer	ACTAGTTGATGCAGAGTTACC
		M3_probe	CTACACCCATTTGGGAAATGCCTTC
N1	140	N1_forward primer	CTTGTTGTGGTAAAAATAGCTTAG
		N1_reverse primer	GGAAGTTTTCATGAGTAAGAGC
		N1_probe	CAATAACTGGCGCAGCAAACATACCATAC

After confirming the positive detection of different bacterial species, the universal 16S rRNA gene primer pair and hydrolysis probe (U16S) were evaluated using qPCR. As anticipated and based on our previous experience, the NTCs, which do not contain template DNA but do contain primers specific to a conserved region in the 16 rRNA gene, produced a background threshold cycle (*C_T_*) value that ranged between 31 and 34. Similarly, the usage of the Laboratory Response Network’s universal 16S rRNA gene primer/probe set for the NTCs resulted in an average background *C_T_* value of 33. These background values were present for all the commercially available thermostable DNA polymerase I enzymes tested in the qPCR assays. This background signal was due to the residual carryover of bacterial DNA in the thermostable DNA polymerase I preparations and subsequent amplification of the conserved 16S rRNA gene target. Although the residual 16S rRNA gene carryover in this thermostable enzyme did reduce the signal-to-noise ratio in the U16S qPCR assay and increase the LOD (Table 5), the conservation of this target in bacteria outweighs this drawback by providing valuable information about the presence of bacteria. Accordingly, the presence of carryover bacterial DNA in the thermostable DNA polymerase I required a *C_T_* value to be less than 31 in the U16S qPCR assay to detect the presence of bacteria or bacterial DNA in the sample.

**TABLE 5:** F. tularensis subspecies and/or subtype singleplex qPCR differentiation assay, chromosomal target, and limit of detection

Singleplex qPCR assay^*a*^	Organism detected	Chromosomal target (locus tag)^*b*^	LOD^*c*^
U16S	Bacteria	16S rRNA gene	∼0.1^*d*^
4Pan	F. tularensis subsp. *tularensis* (type A)	*ostA1* (FTT_0467)	3
	F. tularensis subsp. *holarctica* (type B)		
	F. tularensis subsp. *mediasiatica*		
	F. tularensis subsp. *novicida*		
3Pan	F. tularensis subsp. *tularensis* (type A)	Hypothetical gene (FTL_1858)	5
	F. tularensis subsp. *holarctica* (type B)		
	F. tularensis subsp. *mediasiatica*		
A1d	F. tularensis subsp. *tularensis* subtype A.I	Hypothetical gene (FTT_0516)	7
A2c	F. tularensis subsp. *tularensis* subtype A.II	*mviN* (FTW_1702)	5
B2	F. tularensis subsp. *holarctica* (type B)	Hypothetical gene (FTS_0806)	5
M3	F. tularensis subsp. *mediasiatica*	Hypothetical gene (FTM_1104)	2
N1	F. tularensis subsp. *novicida*	Metabolite H+ symporter (FTN_0003)	3

a Hydrolysis probes used in the singleplex qPCR assays were labeled with the fluorophore 5′ FAM and 3′ quencher BHQ1.

b Locus tag prefix with associated F. tularensis strain: FTT, SCHU S4 (subtype A.I); FTW, WY96-3418 (subtype A.II); FTL, LVS (attenuated type B); FTS, FSC200 (type B); FTM, FSC147 (F. tularensis subsp. *mediasiatica*); FTN, U112 (F. tularensis subsp. *novicida*).

c Units are fg except where indicated.

d Unit is pg.

The optimized forward primer, reverse primer, and hydrolysis probe concentrations for the singleplex U16S qPCR assay were determined to be 0.5 μM, 0.5 μM, and 0.2 μM, respectively. The LOD for the singleplex U16S qPCR assay was determined to be approximately 0.1 pg of bacterial DNA and was dependent upon the copy number of the 16S rRNA gene (Table 5). The average *C_T_* value obtained in this assay for all the F. tularensis strains and the different bacterial species in the exclusivity panel, including the DNA extracted from ticks lysates, was 24.97. The *C_T_* values in the U16S qPCR assay ranged from 17.46 to 29.98, depending upon the amount of bacterial DNA present and the copy number of the 16S rRNA gene target in the bacterial genome(s). All NTC background *C_T_* values were greater than 31 in these assessments, due to residual bacterial DNA in the thermostable DNA polymerase I. Together, these results confirm that this assay reliably detects the presence of bacteria by targeting a conserved region in the 16S rRNA gene.

### F. tularensis pan singleplex qPCR assay development.

To detect all F. tularensis strains, three potential chromosomal targets were identified by the *in silico* analyses. Primers were designed for these targets and initially used in conventional PCR with the reference strains and the exclusivity test panel. The primer pair with the highest species specificity and sensitivity detected a 116-bp region within the gene encoding *ostA* (SCHU S4 locus tag FTT_0467), which is predicted to express an organic solvent tolerance protein. A hydrolysis probe was then designed to this chromosomal target and tested in qPCR. This primer/probe set was referred to as the 4Pan1 qPCR assay, and the associated nucleotide sequences are shown in Table 4. The optimum forward primer, reverse primer, and hydrolysis probe concentrations were determined to be 0.5 μM, 0.75 μM, and 0.3 μM, respectively. The 4Pan1 qPCR assay detected all of the F. tularensis strains from the four different subspecies in the inclusivity panel, but not any of the bacteria in the exclusivity test panel nor the microbes within the ticks. The NTCs were negative, and the LOD in the singleplex 4Pan1 qPCR assay was determined to be 3 fg, which is equivalent to approximately two genomic copies (Table 5). The average *C_T_* value obtained for the representative F. tularensis strain assessed at the LOD of 3 fg was 35.53. The average *C_T_* value obtained for the inclusivity F. tularensis strains evaluated at 30 fg in duplicate was 32.73 for the A.I strains, 33.75 for the A.II strains, 33.71 for the B strains, 34.74 for the F. tularensis subsp. *mediasiatica* strains, and 32.46 for the F. tularensis subsp. *novicida* strains.

The previously identified contiguous regions in the F. tularensis chromosome, referred to as CR10 and CR16, were assessed *in silico* to identify putative targets for the F. tularensis species-specific pan and/or subspecies-specific qPCR assays. We previously described the use of the CR10 and CR16 primer sets in a conventional PCR assay to detect F. tularensis and differentiate the subspecies and subtypes from each other and near neighbors (15). However, the goal of this study was to develop a rapid qPCR assay that accurately differentiates the subspecies and subtypes from each other and closely related bacteria without the need for visualization of the amplicons in a stained gel and without the need for a complex qPCR scoring matrix.

An open reading frame encoding a hypothetical protein in LVS (locus tag FTL_1858) within the CR16 region was identified as a top candidate for the development of a qPCR pan assay that detects all virulent F. tularensis subspecies (F. tularensis subsp. *tularensis*, F. tularensis subsp. *holarctica*, and F. tularensis subsp. *mediasiatica*) but excludes F. tularensis subsp. *novicida*. The FTL_1858-based primer pair gave the expected results in PCR by producing an 83-bp amplicon for only the three virulent F. tularensis subspecies when tested with the reference F. tularensis strains and exclusivity test panel organisms. Therefore, a hydrolysis probe was designed for this targeted region for use in qPCR. This diagnostic test was referred to as the 3Pan qPCR assay, and the associated primer/probe set nucleotide sequences are shown in Table 4. The optimized forward primer, reverse primer, and hydrolysis probe concentrations in the 3Pan qPCR assay were determined to be 0.5 μM, 0.5 μM, and 0.4 μM, respectively. The 3Pan qPCR assay was then utilized to test the DNA extracted from the inclusivity and exclusivity test organisms, including the environmental ticks. These assessments provided the expected results by detecting the three virulent F. tularensis subspecies in the inclusivity panel but not the non-select agent F. tularensis subsp. *novicida* nor any microbes in the exclusivity test panel. All NTCs were negative, and the LOD for the singleplex 3Pan qPCR assay was determined to be 5 fg, which is equivalent to approximately two to three genomic copies (Table 5). The average *C_T_* value obtained for the representative F. tularensis select agent strain assessed at the LOD of 5 fg was 35.87. The average *C_T_* value for the inclusivity F. tularensis select agent strains evaluated at 50 fg in duplicate was 32.87 for the A.I strains, 32.80 for the A.II strains, 33.57 for the B strains, and 33.60 for the F. tularensis subsp. *mediasiatica* strains.

### Subspecies and subtyping singleplex qPCR assay development.

Next, potential targets specific to each of the F. tularensis subspecies and subtypes were downselected and evaluated with PCR. Seven candidate targets that appeared to be specific to F. tularensis subsp. *tularensis* subtype A.I strains based on the *in silico* analyses were selected for preliminary PCR screening. The primer pair that had the highest subtype A.I specificity amplified a 114-bp sequence within part of an oxidoreductase gene and corresponded to SCHU S4 locus tag FTT_0516. Although the other clades contained the targeted primer-binding sites, the distance between these two regions was over 302,140 bp apart, preventing PCR amplification. A hydrolysis probe along with this primer pair was then utilized in qPCR, and this A.I subtyping test was referred to as the A1d qPCR assay. The primer pair and probe nucleotide sequences for the A1d qPCR assay are shown in Table 4. The optimum forward primer, reverse primer, and hydrolysis probe concentrations in this singleplex assay were determined to be 0.5 μM, 0.5 μM, and 0.2 μM, respectively. The A1d qPCR assay was then used to evaluate the F. tularensis strains in the inclusivity and the exclusivity test panels. These evaluations confirmed that the A1d qPCR assay detected only the F. tularensis A.I strains. The F. tularensis subsp. *tularensis* subtype A.II, F. tularensis subsp. *holarctica*, F. tularensis subsp. *mediasiatica*, and F. tularensis subsp. *novicida* strains, as well as the NTCs and microbes in the exclusivity test panel, including the tick lysates, were all negative. The LOD in the singleplex A1d qPCR assay was determined to be 7 fg, which is equivalent to approximately three genomic copies (Table 5). The average *C_T_* value obtained for the representative F. tularensis A.I strain assessed at the LOD of 7 fg was 37.25. The average *C_T_* value obtained for the inclusivity F. tularensis A.I strains tested at 70 fg in duplicate was 32.82.

To detect the F. tularensis subsp. *tularensis* subtype A.II strains, five chromosomal regions were evaluated in PCR. These initial assessments revealed that a 101-bp region within the *mviN* gene (WY96-3418 locus tag FTW_1702), which encodes an integral membrane protein, had the highest subtype A.II specificity. For subsequent qPCR evaluation, a hydrolysis probe was designed for this targeted region, and this test was referred to as the A2c qPCR assay. The associated primer/probe set sequences for the A2c qPCR assay are shown in Table 4. For this assay, the optimum forward primer, reverse primer, and hydrolysis probe concentrations were determined to be 0.5 μM, 0.25 μM, and 0.3 μM, respectively. Assessment of the A2c qPCR assay with the inclusivity and exclusivity test panels confirmed the specific detection of only the F. tularensis A.II strains. The NTCs were all negative, and the LOD in the singleplex A2c qPCR assay was determined to be 5 fg, which is equivalent to approximately two or three genomic copies (Table 5). The average *C_T_* value obtained for the representative F. tularensis A.II strain evaluated at the LOD of 5 fg was 37.66. The average *C_T_* value obtained for the inclusivity F. tularensis A.II strains assessed at 50 fg in duplicate was 33.24.

To identify F. tularensis subsp. *holarctica* (type B) strains, a region predicted to encode a hypothetical protein in FSC200 (locus tag FTS_0806) was selected for PCR analysis. This associated primer pair amplified the expected 80-bp region in the reference type B strain and did not produce any PCR products for the other F. tularensis subpopulations nor for the exclusivity organisms. Therefore, a hydrolysis probe was designed, and the primer/probe set was evaluated in qPCR. This test was referred to as the B2 qPCR assay, and the associated primer/probe set sequences are shown in Table 4. The optimized forward primer, reverse primer, and hydrolysis probe concentrations for this singleplex assay were determined to be 0.5 μM, 0.5 μM, and 0.2 μM, respectively. The B2 qPCR assay detected only the F. tularensis type B strains in the inclusivity test panel and did not detect any of the other organisms in the exclusivity panel. The NTCs were all negative, and the LOD for the B2 qPCR assay was determined to be 5 fg, which is equivalent to approximately three genomic copies (Table 5). The average *C_T_* value obtained for the representative F. tularensis B strain tested at the LOD of 5 fg was 36.43. The average *C_T_* value obtained for the inclusivity F. tularensis B strains evaluated at 50 fg in duplicate was 33.99.

Three genomic signatures specific to F. tularensis subsp. *mediasiatica* were assessed in PCR to determine the uniqueness of these targeted regions to this clade. Primers to a region within a gene encoding a major facilitator superfamily transporter (FSC147 locus tag FTM_1104) provided the highest specificity and sensitivity. This primer pair produced a 112-bp amplicon. An associated hydrolysis probe was then synthesized for testing in qPCR. This test was referred to as the M3 qPCR assay, and the primer/probe sequences are shown in Table 4. The optimized forward primer, reverse primer, and hydrolysis probe concentrations for this singleplex assay were determined to be 1.0 μM, 0.75 μM, and 0.3 μM, respectively. Evaluation of this primer pair with the associated hydrolysis probe in qPCR verified the specificity of this assay to only the F. tularensis subsp. *mediasiatica* strains and did not detect any of the other F. tularensis subspecies nor any of the exclusivity organisms. The NTCs were all negative, and the LOD for the M3 qPCR assay was determined to be 2 fg, which is equivalent to approximately two genomic copies since there are two chromosomal copies of this target (Table 5). The average *C_T_* value obtained for the representative F. tularensis subsp. *mediasiatica* strain assessed at the LOD of 2 fg was 36.87. The average *C_T_* value obtained for the inclusivity F. tularensis subsp. *mediasiatica* strains tested at 20 fg in duplicate was 34.40.

As anticipated, the highest numbers of specific chromosomal signatures were identified in F. tularensis subsp. *novicida* since this non-select agent subspecies differs the most in genomic content compared with the other three select agent subspecies. Three candidate targets were selected for the initial PCR assessment. The primer pair that amplified a 140-bp region within the gene encoding a metabolite:H+ symporter family protein (U112 locus tag FTN_0003) provided specific and sensitive detection of F. tularensis subsp. *novicida*. Therefore, an associated hydrolysis probe was designed for subsequent qPCR analysis. This singleplex test was referred to as the N1 qPCR assay, and the associated primer/probe set sequences are shown in Table 4. The optimized forward primer, reverse primer, and hydrolysis probe concentrations for this assay were determined to be 1.0 μM, 0.75 μM, and 0.3 μM, respectively. Assessment of this primer/probe set in qPCR was specific to only F. tularensis subsp. *novicida* strains and did not detect any of the other F. tularensis subspecies or exclusivity organisms. The NTCs were all negative, and the LOD for the N1 qPCR assay was determined to be 3 fg, which is equivalent to approximately two genomic copies (Table 5). The average *C_T_* value obtained for a representative F. tularensis subsp. *novicida* strain evaluated at the LOD of 3 fg was 36.34. The average *C_T_* value obtained for the inclusivity F. tularensis subsp. *novicida* strains assessed at 30 fg in duplicate was 33.01.

### Two-tier multiplex qPCR assay development.

To provide critical information on the identity of the microbe in question in a reduced number of tests, a two-tier multiplex approach was designed utilizing seven of the eight above-described qPCR assays. The tier 1 platform included the U16S, 4Pan1, 3Pan, and A1d qPCR assays for the detection of bacteria, all four F. tularensis subspecies, the three virulent F. tularensis subspecies, and the most virulent subtype A.I, respectively. The tier 2 platform included the U16S, A2c, B2, and M3 qPCR assays that would identify the presence of bacteria, F. tularensis subtype A.II, F. tularensis subsp. *holarctica*, and F. tularensis subsp. *mediasiatica*, respectively.

The tier 1 multiplex platform was designed to serve as a single test that would detect the presence of a virulent select agent F. tularensis strain or, conversely, the non-select agent F. tularensis subsp. *novicida*. More specifically, positive detection of the U16S, 4Pan1, 3Pan, and A1d targets indicates that the organism is a hypervirulent F. tularensis A.I strain. Positive detection of only the U16S and 4Pan1 targets and negative results for the 3Pan and A1d targets would identify the bacterial strain as non-select agent F. tularensis subsp. *novicida*. Lastly, a positive result for the U16S, 4Pan1, and 3Pan targets but a negative result for the A1d target would indicate that the bacterial strain is the virulent select agent F. tularensis but not hypervirulent A.I nor non-select agent F. tularensis subsp. *novicida*.

In summary, the tier 1 quadruplex qPCR assay was designed to provide reliable and important information on whether the organism was a bacterial strain, a F. tularensis strain, a select agent F. tularensis strain, a hypervirulent A.I select agent strain, or a non-select agent F. tularensis subsp. *novicida* strain. If needed, the tier 2 multiplex platform would identify the subspecies and/or subtype of the F. tularensis select agent strain that was not an A.I strain nor a non-select agent F. tularensis subsp. *novicida* strain. If the results from the quadruplex assays indicate that the organism is a F. tularensis subsp. *novicida* strain and further confirmation is of interest, the singleplex F. tularensis subsp. *novicida*-specific (N1) qPCR assay could be utilized. Alternatively, the N1 target could be incorporated into either the tier 1 or 2 multiplex qPCR assays, as long as the fluorophore used for this hydrolysis probe is compatible with the other fluorophores and a real-time instrument is used. Evaluation of all the F. tularensis inclusivity strains and the exclusivity test panel in the tier 1 and tier 2 multiplex platforms confirmed the accurate identification of the F. tularensis clade. Table 6 summarizes the F. tularensis subspecies and subtype(s) identified in the tier 1 and tier 2 multiplex platforms, along with the associated qPCR assays.

**TABLE 6:** Summary of F. tularensis subspecies and subtypes identified by tier 1 and tier 2 multiplex platforms^*a*^

Multiplex qPCR platform	qPCR assay target (fluorophore/quencher)	Organism(s) detected	LOD (fg)
Tier 1^*b*^	U16S (Quasar 670/BHQ3)	Bacteria	50
	4Pan1 (TAMRA/BHQ2)	F. tularensis subsp. *tularensis*, F. tularensis subsp. *holarctica*, F. tularensis subsp. *mediasiatica*, F. tularensis subsp. *novicida*	10
	3Pan (CAL Fluor Orange 560/BHQ1)	3 Virulent F. tularensis subspecies (F. tularensis subsp. *tularensis*, F. tularensis subsp. *holarctica*, F. tularensis subsp. *mediasiatica*)	30
	A1d (FAM/BHQ1)	F. tularensis subsp. *tularensis* subtype A.I	30
Tier 1^*c*^	U16S (Quasar 670/BHQ3)	Bacteria	100
	4Pan1 (Texas Red/BHQ2)	F. tularensis subsp. *tularensis*, F. tularensis subsp. *holarctica*, F. tularensis subsp. *mediasiatica*, F. tularensis subsp. *novicida*	10
	3Pan (HEX/BHQ1)	3 Virulent F. tularensis subspecies (F. tularensis subsp. *tularensis*, F. tularensis subsp. *holarctica*, F. tularensis subsp. *mediasiatica*)	30
	A1d (FAM/BHQ1)	F. tularensis subsp. *tularensis* subtype A.I	30
Tier 2^*c*^	U16S (Quasar 670/BHQ3)	Bacteria	50
	A2c (FAM/BHQ1)	F. tularensis subsp. *tularensis* subtype A.II	10
	B2 (Texas Red/BHQ2)	F. tularensis subsp. *holarctica* (type B)	30
	M3 (JOE/BHQ1)	F. tularensis subsp. *mediasiatica*	10

a F. tularensis clades detected and differentiated using various real-time thermocyclers with compatible fluorophore and quencher hydrolysis probes.

b Quadruplex assay utilizing hydrolysis probes with 5′ fluorophores and 3′ quenchers that are compatible with the 7500 Fast Dx real-time PCR system.

c Quadruplex assay utilizing hydrolysis probes with 5′ fluorophores and 3′ quenchers that are compatible with both the 7500 Fast Dx real-time PCR system and the 3M Integrated Cycler.

To ensure that the tier 1 multiplex platform can be reliably utilized on different real-time thermocyclers, the assay was run on the 7500 Fast Dx real-time PCR system and the 3M Integrated Cycler by at least two different individuals, using hydrolysis probes with compatible fluorophores and quenchers. Optimized primer and probe concentrations were determined for these two real-time thermocyclers and are shown in Table 7. LOD assessments for each target in the tier 1 multiplex platform was next performed with genomic DNA from the F. tularensis A.I reference strain (SCHU S4). The LODs with hydrolysis fluorophores and quenchers appropriate for the 7500 Fast Dx real-time PCR system were determined to be 50 fg for the 16S rRNA gene target (U16S), 10 fg for the 4Pan1 target, 30 fg for the 3Pan target, and 30 fg for the A1d target (Table 6). These LODs were determined to be the same when using the 3M Integrated Cycler for the two pan assays (4Pan1 and 3Pan) and the subtype A.I detection assay (A1d); however, the LOD for the 16S rRNA gene target (U16S) was 100 fg instead of 50 fg (Table 6). When genomic DNA from the appropriate F. tularensis inclusivity strains was tested in duplicate at 1 pg, the average *C_T_* values obtained for the U16S, 4Pan1, 3Pan, and A1d targets in the tier 1 multiplex platform were 27.09, 30.24, 34.61, and 35.34, respectively. All NTCs were negative, except for the 16S rRNA gene target (U16S), which had a *C_T_* value higher than 31 due to residual E. coli DNA in the recombinant polymerase utilized.

**TABLE 7:** Optimized hydrolysis probe and primer concentrations for the tier 1 and tier 2 multiplex platforms^*a*^

Multiplex platform	qPCR assay (fluorophore/quencher)	Probe concn (μM)	Forward primer concn (μM)	Reverse primer concn (μM)
Tier 1	U16S (Quasar 670/BHQ3)	0.2	0.5	0.5
	4Pan1 (Texas Red/BHQ2)	0.4	0.5	0.75
	3Pan (HEX/BHQ1)	0.5	0.5	0.5
	A1d (FAM/BHQ1)	0.2	0.5	0.5
Tier 2	U16S (Quasar 670/BHQ3)	0.1	0.5	0.5
	A2c (FAM/BHQ1)	0.3	0.5	0.25
	B2 (Texas Red/BHQ2)	0.6	0.5	0.5
	M3 (JOE/BHQ1)	0.5	1.0	0.75

a The platforms are compatible with both the Applied Biosystems 7500 Fast Dx real-time PCR system and the 3M Integrated Cycler.

The optimized tier 2 primer and probe concentrations with compatible fluorophores for the two real-time thermocyclers were then determined and are shown in Table 7. The LOD for the tier 2 multiplex assay on the 7500 Fast Dx real-time PCR system and the 3M Integrated Cycler were determined to be 50 fg for the 16S rRNA gene target (U16S), 10 fg for subtype A.II (A2c), 30 fg for type B (B2), and 10 fg for F. tularensis subsp. *mediasiatica* (M3), as noted in Table 6. When genomic DNA from the appropriate F. tularensis inclusivity strains was tested in duplicate at 0.5 pg, the average *C_T_* values obtained for the U16S, A2c, B3, and M3 targets in the tier 2 multiplex platform were 28.51, 30.93, 32.91, and 30.98, respectively. All NTCs were negative, except for the U16S target in which the *C_T_* value was again higher than 31.

All of the singleplex qPCR assays and multiplex platforms were assessed for linearity, one of the most important characteristics for evaluating the accuracy of an assay during validation. Ten-fold dilutions of the appropriate F. tularensis genomic DNA resulted in a linear standard curve with R^2^ values of 0.998, 0.999, 0.976, 0.999, 0.998, 0.997, and 1.00 for the singleplex 4Pan1, 3Pan, A1d, A2c, B2, M3, and N1 qPCR assays, respectively. For the tier 1 multiplex platform, the R^2^ values of 0.961, 0.983, 0.998, and 0.996 were obtained for the U16S rRNA gene, 4Pan1, 3Pan, and A1d targets, respectively. The R^2^ values acquired for the tier 2 multiplex platform were 0.982, 1.000, 0.968, and 1.000 for the U16S, A2c, B2, and M3 targets, respectively. Together, these data corroborated that the singleplex qPCR assays and multiplex platforms described in this study provide accurate, specific, and sensitive detection of F. tularensis and, importantly, offers the capability to differentiate the subspecies or subtype for both clinical and environmental applications.

Performing qPCR directly from a human, animal, or environmental specimen would not give consistent results for any PCR-based assay, due to inherent differences in sample constituents, which could inhibit and/or alter the detection of the target of interest. Therefore, the goal of this study was to provide reliable, straightforward, and sensitive assays to identify and differentiate the subspecies and subtypes of F. tularensis, after culturing this organism on appropriate media (e.g., chocolate agar plates). To accurately determine assay sensitivity, genomic DNA isolation was required; however, we have successfully used the described qPCR assays directly from CFUs. More specifically, several CFUs were aseptically transferred to 100 μl of water, thoroughly mixed, and heated to 98°C for 10 minutes, and then approximately 2 μl of this homogenous suspension was used in a 20-μl reaction in the singleplex and multiplex qPCR assays for F. tularensis identification.

### Verification of F. tularensis qPCR assay specificity and the presence of *Francisella*-like organisms in ticks.

To confirm the specific, but sensitive detection of only F. tularensis strains and to verify the presence of *Francisella*-like endosymbionts known to exist in ticks, additional analyses were performed. The average *C_T_* values obtained in the singleplex and multiplex U16S qPCR assays when testing the DNA extracted from tick lysates were 21.87 (ranging from 19.26 to 24.50) and 22.47 (ranging from 19.76 to 25.19), respectively. These low *C_T_* values compared with the respective NTC value (*C_T_*, >31) provided evidence that these environmental specimens contained bacteria.

To verify the presence of *Francisella*-like endosymbionts in the ticks, PCR amplifications were performed using a previously available commercial assay and primers known to amplify target genes in *Francisella*-like bacteria (22–25). For these tests, *F. persica* served as a positive control for a *Francisella*-like endosymbiont (14, 15). These results demonstrated that 9 out of the 19 environmental ticks tested contained *Francisella*-like organisms and all 9 positive results were obtained from a Dermacentor andersoni or Dermacentor variabilis tick (Table 3). These findings confirmed the presence of *Francisella*-like organisms in the majority of *Dermacentor* sp. ticks. Although the Amblyomma americanum and Ixodes scapularis ticks did not produce a positive result with the primers used (Table 3), these ticks may contain other *Francisella*-like organisms that were not targeted for amplification. Therefore, DNA extracted from 4 tick species, which included 25 specimens in total and are shown in Table 3, were included in the exclusivity test panel.

To ensure that no inhibition was occurring in the F. tularensis-specific qPCR assays, DNA from the tick lysates were spiked with the appropriate F. tularensis reference strain for subsequent testing in these analyses. All of the singleplex qPCR assays and the multiplex platforms accurately detected the relevant F. tularensis subspecies and/or subtype, confirming that inhibition was not occurring. Collectively, these results demonstrated that the qPCR assays developed in this study accurately detect and differentiate F. tularensis from other bacteria with shared genomic content, which are often present in ticks.

### Amplicon sequencing for identification confirmation and potential strain information.

To expand the capabilities of the tier 1 multiplex qPCR assay for strain differentiation, the primers to each target in this platform were synthesized with PGM-compatible barcodes and used in conventional PCR. The resulting amplicons were then sequenced on the Ion Torrent PGM system and were analyzed for content. These evaluations and comparisons to other sequences in databases further confirmed F. tularensis clade identity and contributed valuable strain information about the presence of any SNPs, implicating potential relatedness.

To determine if the barcoded primers in the tier 1 multiplex platform affected assay specificity and robustness, 100 pg of genomic DNA from F. tularensis and the exclusivity bacteria was tested. These results demonstrated that the appropriate F. tularensis strains were identified in the 4Pan1, 3Pan, and A1d qPCR assays, even though the primers were barcoded. The *C_T_* values obtained with the barcoded primers versus the unlabeled primers were also compared. The average *C_T_* value obtained for the U16S target in the tier 1 qPCR assay with the barcoded primer pairs was 24.13, in comparison to the average *C_T_* value of 22.18 that was attained with the unlabeled primer pairs for the F. tularensis inclusivity and exclusivity DNA. For the 4Pan1, 3Pan, and A1d targets with the barcoded primer pairs, the average *C_T_* values obtained were 27.09, 27.50, and 28.22 for the appropriate F. tularensis strains, respectively. In comparison, *C_T_* values obtained for the 4Pan1, 3Pan, and 1Ad targets in the multiplex tier 1 platform with unlabeled primers were 23.34, 31.37, and 26.93, respectively. Therefore, the *C_T_* values obtained in general with the barcoded primer pairs were only slightly higher than the values attained with the unlabeled primers, with the exception of the 3Pan target in which these values were marginally lower. In summary, these assessments demonstrated that the tier 1 multiplex platform could be utilized with barcoded primers for subsequent sequencing to further confirm and differentiate F. tularensis strains.

## DISCUSSION

Special precautions are warranted when dealing with an unknown microbe with the potential to be a hypervirulent select agent. Therefore, to rapidly and accurately determine whether the organism is a F. tularensis select agent strain and to reduce the number of tests, two multiplex qPCR assays were developed, specifically the tier 1 and tier 2 platforms. These quadruplex tests were based on the successful capability of the singleplex qPCR assays produced in this study. The tier 1 multiplex platform provides a fast answer to whether the organism in question is conclusively F. tularensis and, if so, if it is (i) a hypervirulent select agent A.I strain, (ii) a strain from one of the other virulent select agent clades (subtype A.II, type B, or F. tularensis subsp. *mediasiatica*), or (iii) a non-select agent strain associated with F. tularensis subsp. *novicida*. If additional subpopulation information is needed to determine the identity of the F. tularensis select agent, which is not a hypervirulent A.I strain, the tier 2 multiplex platform could be used. Together, these two multiplex qPCR assays rapidly and definitively identify F. tularensis and the associated clade with fewer tests than other qPCR assays, and they do not require a complex scoring matrix for interpretation of the results.

The LOD for our previously described differential insertion sequence amplification (DISA) assay using standard PCR conditions was determined to be 40 fg, which is approximately 20 genomic copies of the 1.9-Mbp F. tularensis chromosome (15). Singleplex qPCR assays developed by others to differentiate three of the F. tularensis subspecies obtained LODs that ranged from 25 fg to 250 fg or 12 to 122 genomic copies, and the associated multiplex qPCR assay had LODs between 250 fg to 2.5 pg or 122 to 1,221 genomic copies (16). The LODs obtained for the qPCR assays described in this study demonstrated a substantial improvement in sensitivity. The LOD for the singleplex F. tularensis-specific qPCR assays ranged between 2 fg and 7 fg or 1 and 3 genomic copies, and the LOD for the tier 1 and tier 2 multiplex platforms ranged between 10 fg to 100 fg or 5 to 49 genomic copies. The approximate 5-fold to 10-fold increase in the LOD for the quadruplex qPCR assays compared with the singleplex qPCR assays concurs with the findings of others (16, 29). Since the same qPCR cycling parameters were used in the singleplex and multiplex assays, this reduction in the LOD is most likely due to the multiple copies of the internal control, specifically the conserved region in the 16S rRNA gene, as well as competitive PCR issues with the presence of four targets in a single reaction. Nevertheless, template:*C_T_* plots revealed parallel lines for the multiplex and singleplex qPCR assays, indicating comparable efficiencies.

F. tularensis subtype A.I, subtype A.II, and type B strains are present in North America, and hard ticks are considered one of the major biological vectors for the transmission of tularemia (30). Misleading results due to cross-reactivity with near neighbors and *Francisella*-like endosymbionts known to exist in ticks has occurred (22, 23, 31). The low *C_T_* values obtained in the current study when targeting the conserved region in the 16S rRNA gene of bacteria (U16S target) verified the presence of bacterial DNA in the ticks. Furthermore, the DNA extracted from the ticks was previously tested using an assay known to detect F. tularensis-like endosymbionts, confirming the presence of microbes that shared considerable genomic content with F. tularensis in ticks (15). The exclusivity test panel in the current study included environmental tick lysates that were previously determined to contain DNA from F. tularensis-like organisms (Table 3). Therefore, the data acquired with the qPCR assays described in this study when tested with the DNA extracted from these tick lysates confirmed the specific, but sensitive detection of F. tularensis.

Although PCR-based methods can rapidly identify the pathogen species and subpopulations, these assays often lack strain-level discrimination. Vogler et al. used 23 assays targeting canonical SNPs (canSNPs) to identify F. tularensis and assess phylogeographic associations (32). Seventeen canSNPs were utilized in an allele-specific mismatch amplification mutation assay that coupled GC- and T-rich primer tails, SYBR green dye, and melting curve analysis for SNP genotyping, along with six canSNP allelic discrimination qPCR assays to identify the major groups within F. tularensis. Birdsell et al. further expanded the 6 canSNP assays to 11 canSNP allelic-specific qPCR assays, which could differentiate F. tularensis and the subpopulations if used in a stepwise hierarchical scheme and if parameters were adjusted for some of the tests (17). The false-positive and false-negative results that intermittently occurred in these qPCR assays were attributed to additional SNPs within the primer/probe sites. Svensson and associates determined that the results obtained with the 23 canSNPs and 11 indels were incomplete for the direct detection of F. tularensis subsp. *holarctica* due to the scarcity of pathogen DNA in infected specimens; however, a sufficient number of the targeted markers were identified for isolate characterization in six infected ulcer specimens (33). In a study by Johansson et al., PCR allowed the detection of F. tularensis DNA in 30 (75%) ulcer specimens, whereas bacterial growth of this pathogen after 1 to 3 days in Amies agar with charcoal transport medium was only detected in 25 (62%) of these specimens (34).

The high average nucleotide identity for the different F. tularensis subpopulations that exist in the United States, the presence of *Francisella*-like organisms in the environment, and the broad host range of this fastidious pathogen have complicated both clinical diagnostics and epidemiological investigations. According to the Council of State and Territorial Epidemiologists, the detection of F. tularensis in a specimen by PCR is regarded as supportive laboratory evidence of an infection by this pathogen, whereas culturing when there was or was not a 4-fold rise in antibody titer to antigens from this pathogen is considered confirmatory for F. tularensis. Furthermore, diagnostic microbiology laboratories in hospitals within the United States are required to use Food and Drug Administration (FDA)- and CDC-approved tests to determine the identity of the pathogen present in patient specimens. These tests include aseptically culturing the organism on various types of media in a biosafety cabinet and subsequent incubation for growth. After colonies are apparent, which is typically 2 days for F. tularensis, several CFUs are analyzed using CDC-approved qPCR assays for species confirmation. However, these currently approved assays cannot conclusively differentiate select agent F. tularensis strains from the F. tularensis subsp. *novicida* or *Francisella*-like endosymbionts, unlike the singleplex and multiplex qPCR assays described in the current study.

In summary, the tier 1 and tier 2 quadruplex assays offer a rapid and sensitive method in two qPCR tests for the accurate genotyping of this species, facilitating treatment decisions and the identification of the point source during a tularemia outbreak. These qPCR assays may be used in tandem with DNA sequencing for further confirmation and strain-based characterization without altering specificity. To prevent an infection from worsening and prior to confirming the identity of the pathogen, patients presenting clinical symptoms indicative of tularemia are put on a broad-spectrum antibiotic regimen. Therefore, additional studies are needed to develop methods that directly detect pathogens in clinical specimens without any inconsistencies to rapidly and reliably diagnose tularemia. Diagnostic tools will undoubtedly continue to evolve by comprehensively characterizing the disease-causing pathogen(s) in an expeditious manner for enhanced antibiotic stewardship, particularly for multidrug-resistant organisms.
